# The effect of postoperative adjuvant chemotherapy on survival outcomes in patients with early stage oral squamous cell carcinoma

**DOI:** 10.1038/s41598-025-11565-y

**Published:** 2025-07-25

**Authors:** Yungang He, Xueqin Gong, Hongzhu Tao, Yuehang He, Yuxuan Cao, Yaowen Zhang, Xi Tang, Jia Liu

**Affiliations:** 1 Department of Head and Neck Oncology, Chongqing University Cancer Hospital, Chongqing, China; 2 Department of Radiology, Chongqing University Cancer Hospital, Chongqing, China; 3 Vocational School, Chongqing Zhixing Health School, Chongqing, China; 4 Chongqing College of Traditional Chinese Medicine, Chongqing, China; 5 Chongqing Key Laboratory for Intelligent Oncology in Breast Cancer (iCQBC), Chongqing University Cancer Hospital, Chongqing, China; 6 Cancer Center, Faculty of Health Sciences, University of Macau, Macau, SAR, China; 7 Department of Nuclear Medicine, Chongqing University Cancer Hospital, Chongqing, China; 8 Department of Head and Neck Oncology, Chongqing University Cancer Hospital, No.181 Hanyu Road, Shapingba district, Chongqing, 400000 China

**Keywords:** Oral squamous cell carcinoma, Postoperative, Adjuvant chemotherapy, Survival outcomes, Cancer, Diseases

## Abstract

The role of adjuvant chemotherapy (Adjuvant CT) in early-stage oral squamous cell carcinoma (OSCC) remains unclear; this study aimed to fill this gap by examining the association between Adjuvant CT and survival outcomes in patients with early-stage OSCC who underwent surgery. A retrospective cohort study was conducted to examine patients diagnosed with early-stage OSCC who underwent surgery between 2004 and 2021. Data were obtained from the Surveillance, Epidemiology, and End Results (SEER) database for evaluating the survival outcomes of individuals who received Adjuvant CT compared to those who did not. To assess the relationship between Adjuvant CT and survival outcomes, statistical methods, including Cox regression analysis, propensity score matching (PSM), and Kaplan-Meier survival analysis were utilized. A total of 18,532 patients were included, with 2,098 (11.3%) receiving Adjuvant CT. Kaplan-Meier analysis showed that age, sex, tumor site, income level, pathological grade, pT status, neck dissection and radiation were associated with prognosis (*P* < 0.05). Notably, Adjuvant CT was associated with worse overall survival (OS) and disease-specific survival (DSS) compared to no chemotherapy (5-year OS: 63.1% vs. 72.9%; 10-year OS: 47.6% vs. 57.8%, *P* < 0.0001; 5-year DSS: 71.3% vs. 85.3%; 10-year DSS: 61.6% vs. 81.7%, *P* < 0.0001). Consistently, after PSM, Adjuvant CT did not improve outcomes in patients with early-stage OSCC (DSS: *P* = 0.0001; OS:*P* = 0.00066). Cox regression analysis indicated no overall survival benefit from Adjuvant CT in early-stage OSCC. However, subgroup analysis suggested potential benefits for patients with poorly differentiated tumors. Our study revealed that postoperative Adjuvant CT provides minimal survival benefits for early-stage OSCC patients, though subgroup signals observed. Although the result of the subgroup analysis show that Adjuvant CT may benefit those with poorly differentiated tumors, further research is needed to identify specific subgroups that may benefit.

**Supplementary Information:**

The online version contains supplementary material available at 10.1038/s41598-025-11565-y.

## Introduction

Oral squamous cell carcinoma (OSCC) is one of the most prevalent malignancies in the head and neck region, ranking second among all head and neck cancers^1^. In China, its incidence is surpassed only by nasopharyngeal carcinoma, constituting 28.1% of all head and neck malignant tumors^2^. OSCC is a highly malignant neoplasm with an approximate 5-year survival rate of 60%^3^. Early-stage OSCC (stage I-II, pT1-2N0M0) typically exhibits a more favorable prognosis compared to advanced-stage OSCC^4,5^. The 5-year survival rate for patients diagnosed at stages I or II is approximately 69.7%^6^^[,7^. Nevertheless, despite the generally favorable prognosis for early-stage OSCC, overall survival rates remain suboptimal, and patients who experience early recurrence often face significant challenges.

According to the most recent National Comprehensive Cancer Network (NCCN) guidelines, surgery remains the primary treatment modality for early-stage OSCC^7–9^. For patients who are either unwilling to undergo surgery or have medical conditions that contraindicate surgical intervention, radiotherapy is recommended, especially for those with high-risk pathological features^10^. While radiotherapy may partially substitute for surgery and enhance local control rates, it is associated with notable adverse effects such as oral mucosal injury, cervical scarring, restricted mouth opening, and impaired taste sensation^11,12^. Moreover, due to complications like vascular damage in the neck region and compromised wound healing capabilities, surgical intervention for recurrent cases may present significant challenges^13,14^.

Chemotherapy plays a multifaceted role in the management of various malignant tumors by inhibiting tumor cell proliferation, inducing apoptosis, and enhancing cancer cell sensitivity to other therapeutic modalities. In addition to directly targeting rapidly dividing cells, chemotherapy could alleviate symptoms and improve quality of life in patients with advanced-stage cancer^15,16^. In the management of OSCC, the application of chemotherapy (including neoadjuvant and adjuvant regimens) is progressively expanding^17,18^particularly in certain specific clinical contexts, such as cases with positive surgical margins, lymph node metastasis, or highly invasive tumor biology. Neoadjuvant CT aims to reduce tumor size, facilitate surgical resection, and increase the rate of negative surgical margins. For instance, clinical research has demonstrated that neoadjuvant CT significantly enhances pathological complete response rates and reduces local recurrence rates^19–21^. Furthermore, the adverse effects of radiotherapy on the oral mucosa, oral function, and the shoulder and neck regions tend to be prolonged, while those of chemotherapy typically resolve as the drugs are metabolized.

Considering the potential benefits of chemotherapy for OSCC, it is imperative to investigate whether Adjuvant CT could enhance the prognosis of patients following early-stage OSCC surgery. While some studies have demonstrated that chemotherapy has a certain efficacy in treating locally advanced-stage OSCC, research specifically addressing early-stage OSCC remains limited. A comprehensive review of the PubMed database indicates a paucity of literature on the use of chemotherapy in early-stage OSCC, highlighting significant research gaps in this area. Further investigation into whether Adjuvant CT can improve survival outcomes for early-stage OSCC patients will contribute to refining clinical treatment strategies and providing more precise therapeutic options.

This study concentrated on patients diagnosed with early-stage OSCC classified as pathological stage T1-2N0M0 (pT1-2N0M0). A large-scale retrospective cohort analysis was performed utilizing the internationally recognized SEER database to establish a robust clinical evidence foundation. The findings indicated that Adjuvant CT did not significantly enhance the overall prognosis for early-stage OSCC patients. However, the observation that Adjuvant CT might confer a potential protective benefit in poorly differentiated subpopulations highlights the necessity for future in-depth investigations into specific subpopulations.

## Results

### Clinicopathologic characteristics

A total of 18,532 eligible patients with early-stage OSCC were included in the cohort analysis. Among these patients, 7,124 (38.4%) were under 60 years of age, while 11,408 (61.6%) were 60 years or older. The majority of patients were male, comprising 11,730individuals (63.3%). In terms of ethnicity, White constituted the largest proportion, with 16,125 patients (87.0%). Regarding the primary site of cancer, tongue cancer was the most prevalent, accounting for 10,481cases (56.6%), followed by lip cancer with 3,269cases (17.6%). Marital status indicated that the majority of patients were married, representing 10,353individuals (55.9%). Additionally, the largest proportion of patients had moderately differentiated tumors, comprising 7,935 cases (42.8%).

In this study, 16,434 patients (88.7%) underwent surgery alone, whereas 2,098 patients (11.3%) received Adjuvant CT. The follow-up period for the population ranged from 0 to 215 months, and the median follow-up time was 46 months (interquartile range [IQR]: 19.0, 97.0). A total of 2,688 patients (14.4%) died from OSCC (available for DSS analysis), and a total of 5,910 deaths (31.9%) were available for OS analysis. Table 1 provided a comprehensive summary of the clinicopathological characteristics of the study population, highlighting significant intergroup differences (*P* < 0.05).

To compare the efficacy of the two treatment modalities in a balanced manner, we employed the 1:1 PSM method to construct a target population with more homogeneous patient characteristics. After PSM, 1,593 patients were selected from each group and enrolled in the new treatment groups (as detailed in Table 2).

**Table 1:** Demographic and clinical characteristics before PSM.

Variables	Total (*n* = 18532)	Surgery alone (*n* = 16434)	Adjuvant CT (*n* = 2098)	*P*-value
Age, n (%)				0.867
< 60 years	7124 (38.4)	6321 (38.5)	803 (38.3)	
≥ 60 years	11,408 (61.6)	10,113 (61.5)	1295 (61.7)	
Sex, n (%)				< 0.001
Female	6802 (36.7)	6405 (39)	397 (18.9)	
Male	11,730 (63.3)	10,029 (61)	1701 (81.1)	
Site, n (%)				< 0.001
Lip	3269 (17.6)	3235 (19.7)	34 (1.6)	
Tongue	10,481 (56.6)	8722 (53.1)	1759 (83.8)	
Gum	1154 (6.2)	1108 (6.7)	46 (2.2)	
Floor of mouth	1477 (8.0)	1416 (8.6)	61 (2.9)	
Palate	659 (3.6)	572 (3.5)	87 (4.1)	
Other	1492 (8.1)	1381 (8.4)	111 (5.3)	
Grade, n (%)				< 0.001
Well	5176 (27.9)	5032 (30.6)	144 (6.9)	
Moderately	7935 (42.8)	7331 (44.6)	604 (28.8)	
Poorly	2287 (12.3)	1757 (10.7)	530 (25.3)	
NA	3134 (16.9)	2314 (14.1)	820 (39.1)	
Race, n (%)				< 0.001
White	16,125 (87.0)	14,258 (86.8)	1867 (89)	
Black	666 (3.6)	540 (3.3)	126 (6)	
Others	1517 (8.2)	1422 (8.7)	95 (4.5)	
NA	224 (1.2)	214 (1.3)	10 (0.5)	
Marital status, n (%)				< 0.001
Married	10,353 (55.9)	9052 (55.1)	1301 (62)	
Single	2860 (15.4)	2536 (15.4)	324 (15.4)	
Other	3891 (21.0)	3493 (21.3)	398 (19)	
NA	1428 (7.7)	1353 (8.2)	75 (3.6)	
Residence, n (%)				0.063
Urban	15,837 (85.5)	14,004 (85.2)	1833 (87.4)	
Rural	2680 (14.5)	2417 (14.7)	263 (12.5)	
NA	15 (0.1)	13 (0.1)	2 (0.1)	
Income, n (%)				0.002
< 50,000	1329 (7.2)	1192 (7.3)	137 (6.5)	
50,000–75,000	6793 (36.7)	6093 (37.1)	700 (33.4)	
> 75,000	10,408 (56.2)	9147 (55.7)	1261 (60.1)	
NA	2 (0.0)	2 (0)	0 (0)	
pT status				< 0.001
pT1	12,359 (66.7)	11,454 (69.7)	905 (43.1)	
pT2	6173 (33.3)	4980 (30.3)	1193 (56.9)	
Neck dissection, n (%)				< 0.001
No	11,887 (64.1)	10,262 (62.4)	1625 (77.5)	
Yes	6645 (35.9)	6172 (37.6)	473 (22.5)	
Radiation, n (%)				< 0.001
No	13,512 (72.9)	13,398 (81.5)	114 (5.4)	
Yes	5020 (27.1)	3036 (18.5)	1984 (94.6)	
Survival.months	46.0 (19.0, 97.0)	52.0 (20.0, 102.0)	26.0 (13.0, 45.0)	< 0.001
DSS, n (%)				< 0.001
Alive or dead of other cause	15,864 (85.6)	14,218 (86.5)	1646 (78.5)	
Dead(attributable to the cancer)	2668 (14.4)	2216 (13.5)	452 (21.5)	
OS, n (%)				0.1
Alive	12,622 (68.1)	11,160 (67.9)	1462 (69.7)	
Dead	5910 (31.9)	5274 (32.1)	636 (30.3)	

DSS: disease-specific survival; OS: overall survival; NA: Not Available; Adjuvant CT: adjuvant chemotherapy.

**Table 2:** Demographic and clinical characteristics after PSM.

Variables	Total (*n* = 3186)	0 (*n* = 1593)	1 (*n* = 1593)	*P*-value
Age, n (%)				0.713
< 60years	1174 (36.8)	592 (37.2)	582 (36.5)	
≥ 60years	2012 (63.2)	1001 (62.8)	1011 (63.5)	
Sex, n (%)				0.769
Female	739 (23.2)	366 (23)	373 (23.4)	
Male	2447 (76.8)	1227 (77)	1220 (76.6)	
Site, n (%)				0.326
Lip	85 (2.7)	51 (3.2)	34 (2.1)	
Tongue	2480 (77.8)	1221 (76.6)	1259 (79)	
Gum	87 (2.7)	41 (2.6)	46 (2.9)	
Floor of mouth	133 (4.2)	72 (4.5)	61 (3.8)	
Palate	174 (5.5)	89 (5.6)	85 (5.3)	
Other	227 (7.1)	119 (7.5)	108 (6.8)	
Grade, n (%)				0.066
Well	469 (14.7)	220 (13.8)	249 (15.6)	
Moderately	1698 (53.3)	835 (52.4)	863 (54.2)	
Poorly	1019 (32.0)	538 (33.8)	481 (30.2)	
Race, n (%)				0.799
White	2805 (88.0)	1403 (88.1)	1402 (88)	
Black	187 (5.9)	90 (5.6)	97 (6.1)	
Others	194 (6.1)	100 (6.3)	94 (5.9)	
Marital status, n (%)				0.69
Married	1974 (62.0)	984 (61.8)	990 (62.1)	
Single	529 (16.6)	273 (17.1)	256 (16.1)	
Other	683 (21.4)	336 (21.1)	347 (21.8)	
Residence, n (%)				0.794
Urban	2761 (86.7)	1383 (86.8)	1378 (86.5)	
Rural	425 (13.3)	210 (13.2)	215 (13.5)	
Income, n (%)				0.375
< 50,000	384 (12.1)	201 (12.6)	183 (11.5)	
50,000–75,000	941 (29.5)	480 (30.1)	461 (28.9)	
> 75,000	1861 (58.4)	912 (57.3)	949 (59.6)	
pT status				0.375
pT1	1491 (46.8)	733 (46)	758 (47.6)	
pT2	1695 (53.2)	860 (54)	835 (52.4)	
Neck dissection, n (%)				0.699
No	2230 (70.0)	1110 (69.7)	1120 (70.3)	
Yes	956 (30.0)	483 (30.3)	473 (29.7)	
Radiation, n (%)				0.588
No	236 (7.4)	122 (7.7)	114 (7.2)	
Yes	2950 (92.6)	1471 (92.3)	1479 (92.8)	
Survival.months	25.0 (12.0, 54.0)	35.0 (15.0, 80.0)	19.0 (10.0, 36.0)	< 0.001
DSS, n (%)				0.806
Alive or dead of other cause	2388 (75.0)	1197 (75.1)	1191 (74.8)	
Dead(attributable to the cancer)	798 (25.0)	396 (24.9)	402 (25.2)	
OS, n (%)				< 0.001
Alive	1975 (62.0)	930 (58.4)	1045 (65.6)	
Dead	1211 (38.0)	663 (41.6)	548 (34.4)	

### Survival analysis

Survival analysis was employed to estimate the likelihood of survival within a specified time frame, utilizing the Kaplan-Meier (KM) curve as a fundamental tool.

**Fig. 1:**
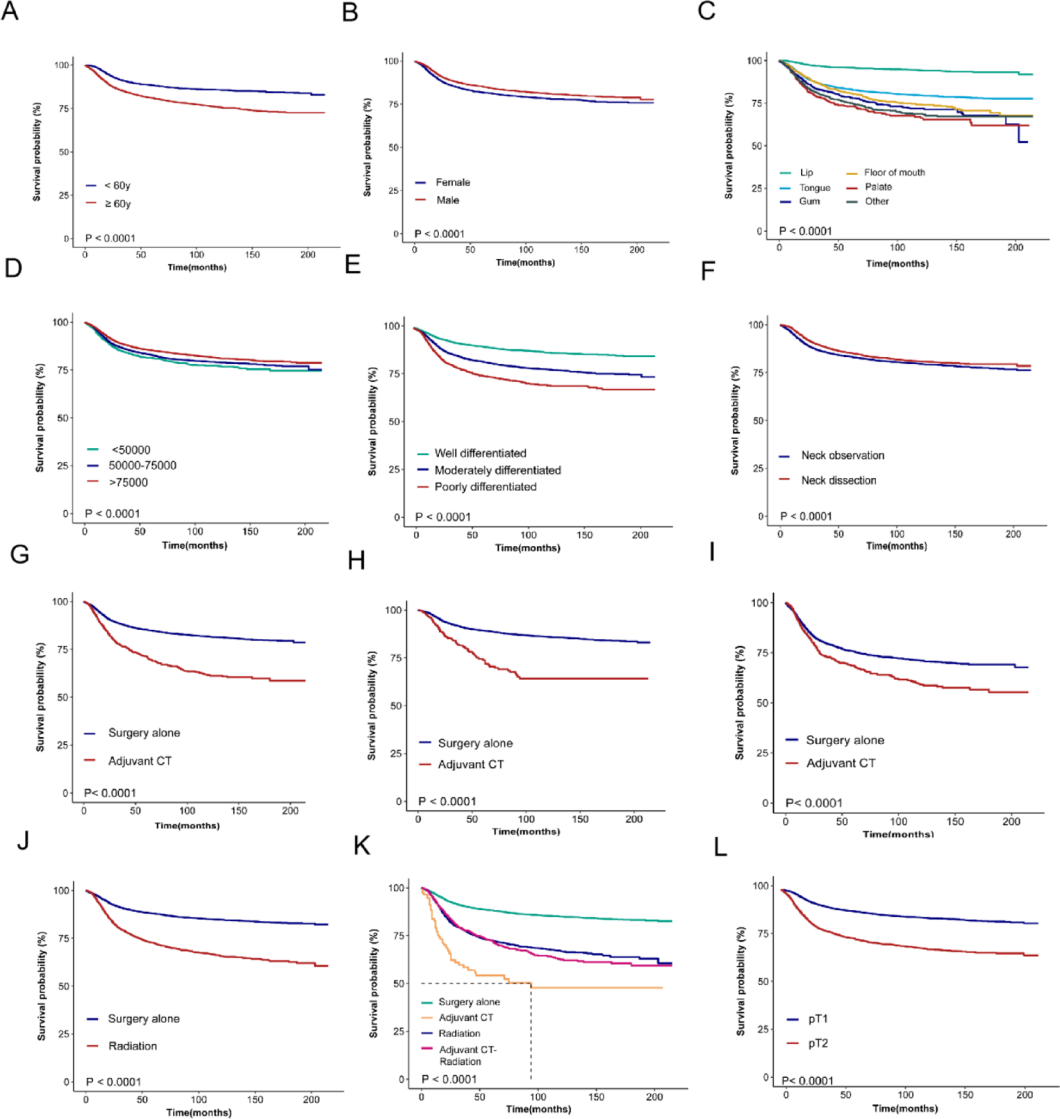
Survival analysis of DSS on patients with early-stage OSCC before PSM: (**A**) age(*P* < 0.0001), (**B**) sex(*P* < 0.0001), (**C**) tumor site(*P* < 0.0001), (**D**) income(*P* < 0.0001), (**E**) pathological grade(*P* < 0.0001), (**F**)neck dissection(*P* < 0.0001), (**G**) adjuvant CT in early-stage (*P* < 0.0001), (**H**) adjuvant CT in stage I (*P* < 0.0001), (**I**) adjuvant CT in stage II (*P* < 0.0001), (**J**) radiation (*P* < 0.0001), (**K**) different treatment groups (*P* < 0.0001;specifically, Adjuvant CT vs. Surgery alone: *P* < 0.0001;Adjuvant CT-Radiation vs. Radiation: *P* = 0.65), and (**L**) pT status(*P* < 0.0001).

**Fig. 2:**
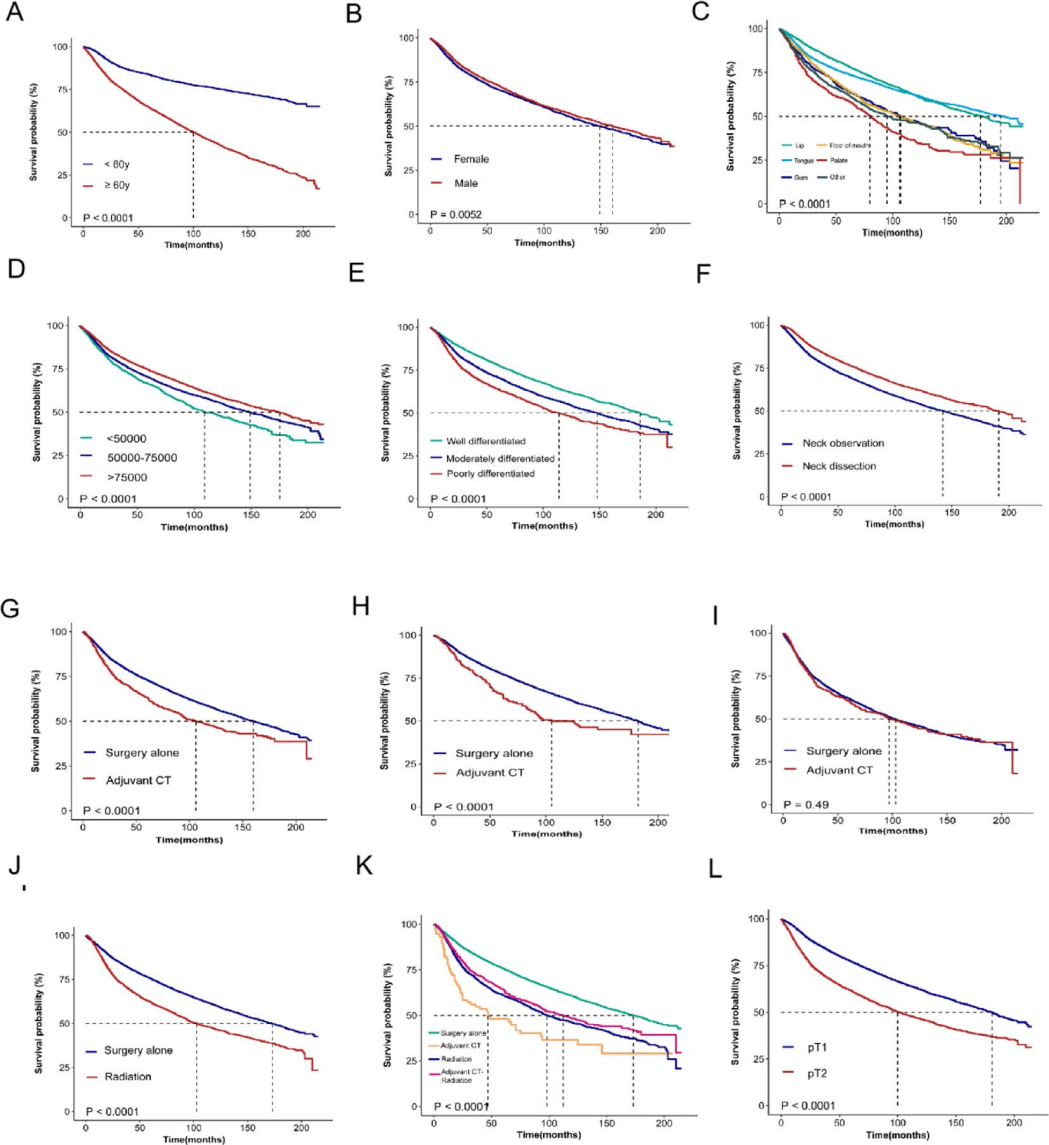
Survival analysis of OS on patients with early-stage OSCC before PSM: (**A**) age (*P* < 0.0001), (**B**) sex(*P* = 0.0052), (**C**) tumor site(*P* < 0.0001), (**D**) income(*P* < 0.0001), (**E**) pathological grade(*P* < 0.0001), (**F**) neck dissection(*P* < 0.0001), (**G**) adjuvant CT in early-stage (*P* < 0.0001), (**H**) adjuvant CT in stage I(*P* < 0.0001), (**I**) adjuvant CT in stage II(*P* = 0.49), (**J**) radiation(*P* < 0.0001), (**K**) different treatment groups (*P* < 0.0001;specifically, Adjuvant CT vs. Surgery alone: *P* < 0.0001;Adjuvant CT-Radiation vs. Radiation: *P* = 0.029), and (**L**) pT status(*P* < 0.0001).

**Fig. 3:**
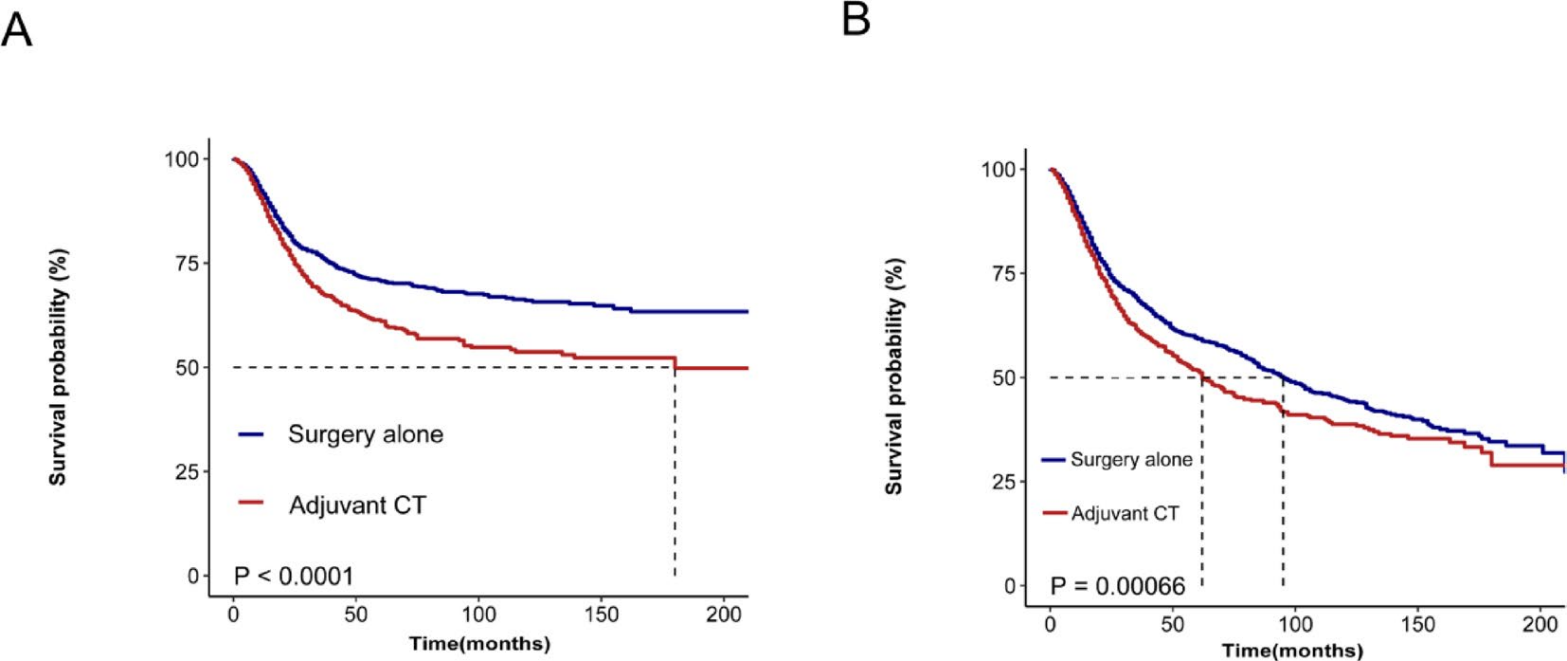
Survival analysis of Adjuvant CT on patients with early-stage OSCC after PSM: (**A**) DSS: disease-specific survival; (**B**) OS: overall survival.

In the DSS analysis, younger age (< 60 years, *P* < 0.0001), male sex (*P* < 0.0001), lip or tongue lesions (*P* < 0.0001), high income (*P* < 0.0001), well-differentiated tumors (*P* < 0.0001), p T1 status (*P* < 0.0001) and neck dissection (*P* < 0.0001) were associated with better outcomes. However, Adjuvant CT and Radiation significantly reduced DSS in early-stage OSCC (*P* < 0.0001), with consistent results of Adjuvant CT in stage I (*P* < 0.0001) and stage II (*P* < 0.0001) (Fig. 1). The results of OS analysis mirrored those of DSS across various factors, with significant associations observed for age group (*P* < 0.0001), sex (*P* = 0.0052), tumor site (*P* < 0.0001), income (*P* < 0.0001), pathological grade (*P* < 0.0001), p T1 status (*P* < 0.0001) and neck dissection status (*P* < 0.0001). Notably, Adjuvant CT and Radiation was associated with reduced OS in early-stage OSCC (*P* < 0.0001), with consistent results of Adjuvant CT in stage I disease (*P* < 0.0001), but it had no significant impact on OS in stage II disease (*P* = 0.49) (Fig. 2). After PSM, Adjuvant CT remained a risk factor for early-stage OSCC in the postoperative period (Fig. 3).

### Cox regression analysis

To evaluate the impact of various individual factors on survival outcomes (DSS and OS), we employed Cox univariate regression analysis, which included age, sex, tumor site, pathological grade, race, marital status, residence, income, pT status, neck dissection, radiation, and Adjuvant CT. Preliminary findings indicated that Adjuvant CT might serve as a significant risk factor for both DSS (*P* < 0.001) and OS (*P* < 0.001) (Table 3). After PSM, the adverse effects of Adjuvant CT remained consistent (Table 4).The association between Adjuvant CT and survival outcomes (DSS and OS) was analyzed using a series of Cox multivariate models, adjusting for various potential confounders (Table 5). For DSS analysis, the unadjusted model (Model 0) showed a hazard ratio (HR) of 2.13(95% CI: 1.92–2.36, *P* < 0.001), indicating a significantly higher risk of disease-specific mortality in the Adjuvant CT group compared to the surgery-alone group. After adjusting for age, sex, and race (Model 1), the HR increased slightly to 2.21(95% CI: 1.99–2.45, *P* < 0.001). Further adjustment for income, marital status, and residence (Model 2) resulted in a slightly higher HR of 2.22(95% CI: 2–2.46, *P* < 0.001). In Model 3, which included tumor site, grade, and pT status in addition to the variables in Model 2, the HR was 1.43(95% CI: 1.28–1.6, *P* < 0.001). Despite a slight decrease in HR compared to Model 2, the association remained statistically significant. For OS analysis, the Model 0 showed an HR of 1.41 (95% CI: 1.29–1.53, *P* < 0.001), indicating a significantly higher risk of overall mortality in the Adjuvant CT group. After adjusting for age, sex, and race, the HR remained at 1.42 (95% CI: 1.31–1.55, *P* < 0.001). Further adjustment for income, marital status, and residence resulted in a slightly higher HR of 1.43 (95% CI: 1.32–1.56, *P* < 0.001). In Model 3, which included tumor site, grade, and pT status, the HR was1.16 (95% CI: 1.06–1.27, *P* < 0.001),consistently indicating an adverse association between Adjuvant CT and overall mortality.

**Table 3:** Univariate analysis identifying prognostic factors for OS and DSS before PSM.

Variables	Diseases-specific survival		Overall survival	
	HR (95%CI)	*P*-value	HR (95%CI)	*P*-value
Age				
< 60years	Ref.		Ref.	
≥ 60years	1.81 (1.66,1.96)	< 0.001	2.86 (2.69,3.03)	< 0.001
Sex				
Female	Ref.		Ref.	
Male	0.82 (0.75,0.88)	< 0.001	0.93 (0.88,0.98)	0.005
Tumor site				
Lip	Ref.		Ref.	
Tongue	4.04 (3.4,4.8)	< 0.001	1.08 (1.01,1.16)	0.029
Gum	5.68 (4.61,7)	< 0.001	1.65 (1.48,1.84)	< 0.001
Floor of mouth	4.94 (4.04,6.03)	< 0.001	1.67 (1.51,1.84)	< 0.001
Palate	7.09 (5.66,8.87)	< 0.001	2.15 (1.9,2.43)	< 0.001
Other	6.45 (5.3,7.85)	< 0.001	1.78 (1.61,1.97)	< 0.001
Grade				
Well	Ref.		Ref.	
Moderately	1.92 (1.74,2.11)	< 0.001	1.33 (1.26,1.42)	< 0.001
Poorly	2.74 (2.44,3.07)	< 0.001	1.68 (1.56,1.81)	< 0.001
Race				
White	Ref.		Ref.	
Black	1.78 (1.51,2.09)	< 0.001	1.43 (1.27,1.62)	< 0.001
Other	1.03 (0.91,1.19)	0.545	0.78 (0.71,0.87)	< 0.001
Marital status				
Married	Ref.		Ref.	
Single	1.26 (1.14,1.4)	< 0.001	1.15 (1.07,1.24)	< 0.001
Other	1.82 (1.67,1.98)	< 0.001	2.05 (1.94,2.17)	< 0.001
Residence				
Urban	Ref.		Ref.	
Rural	1.02 (0.91,1.13)	0.774	1.16 (1.09,1.24)	< 0.001
Income				
< 50,000	Ref.		Ref.	
50,000–75,000	0.85 (0.74,0.97)	0.056	0.83 (0.76,0.92)	< 0.001
> 75,000	0.72 (0.63,0.82)	< 0.001	0.7 (0.64,0.77)	< 0.001
pT status				
pT1	Ref.		Ref.	
pT2	2.46 (2.28,2.66)	< 0.001	1.77 (1.68,1.86)	< 0.001
Neck dissection				
No	Ref.		Ref.	
Yes	0.85 (0.78,0.92)	< 0.001	0.74 (0.7,0.79)	< 0.001
Radiation				
No	Ref.		Ref.	
Yes	2.43 (2.25,2.62)	< 0.001	1.58 (1.49,1.67)	< 0.001
Adjuvant CT				
No	Ref.		Ref.	
Yes	2.13 (1.92,2.36)	< 0.001	1.41 (1.29,1.53)	< 0.001

**Table 4:** Univariate analysis identifying prognostic factors for OS and DSS after PSM.

Variables	Diseases-specific survival		Overall survival	
	HR (95%CI)	*P*-value	HR (95%CI)	*P*-value
Age				
< 60years	Ref.		Ref.	
≥ 60years	1.36 (1.17,1.57)	< 0.001	1.73 (1.53,1.96)	< 0.001
Sex				
Female	Ref.		Ref.	
Male	0.69 (0.6,0.8)	< 0.001	0.84 (0.74,0.95)	0.006
Tumor site				
Lip	Ref.		Ref.	
Tongue	3.13 (1.62,6.06)	< 0.001	1.52 (1.05,2.21)	0.026
Gum	7.23 (3.54,14.76)	< 0.001	3.02 (1.94,4.7)	< 0.001
Floor of mouth	3.54 (1.72,7.25)	< 0.001	2 (1.31,3.07)	0.001
Palate	4.23 (2.09,8.53)	< 0.001	2.06 (1.35,3.13)	< 0.001
Other	5.13 (2.59,10.16)	< 0.001	2.49 (1.67,3.72)	< 0.001
Grade				
Well	Ref.		Ref.	
Moderately	0.87 (0.71,1.05)	0.147	0.82 (0.7,0.95)	0.01
Poorly	0.85 (0.68,1.05)	0.131	0.81 (0.69,0.97)	0.019
Race				
White	Ref.		Ref.	
Black	1.38 (1.06,1.79)	0.017	1.34 (1.08,1.66)	0.009
Other	1.18 (0.91,1.54)	0.211	1.0054 (0.7998,1.2638)	0.963
Marital status				
Married	Ref.		Ref.	
Single	1.24 (1.03,1.5)	0.02	1.19 (1.02,1.39)	0.025
Other	1.34 (1.13,1.58)	< 0.001	1.5 (1.32,1.72)	< 0.001
Residence				
Urban	Ref.		Ref.	
Rural	1.13 (0.93,1.38)	0.205	1.11 (0.95,1.31)	0.184
Income				
< 50,000	Ref.		Ref.	
50,000–75,000	0.96 (0.78,1.19)	0.735	0.9 (0.76,1.07)	0.242
> 75,000	0.7 (0.57,0.85)	< 0.001	0.7 (0.6,0.83)	< 0.001
pT status				
pT1	Ref.		Ref.	
pT2	1.87 (1.62,2.17)	< 0.001	1.64 (1.46,1.84)	< 0.001
Neck dissection				
No	Ref.		Ref.	
Yes	0.66 (0.57,0.78)	< 0.001	0.59 (0.52,0.68)	< 0.001
Radiation				
No	Ref.		Ref.	
Yes	1.02 (0.8,1.32)	0.853	1.09 (0.89,1.34)	0.419
Adjuvant CT				
No	Ref.		Ref.	
Yes	1.41 (1.23,1.62)	< 0.001	1.22 (1.09,1.37)	< 0.001

**Table 5:** Multivariate analysis assessing independent prognostic factors for OS and DSS.

	Disease- specific survival		Overall survival	
	HR((95%CI)	*P-*value	HR((95%CI)	*P-*value
Model 0	2.13 (1.92 ~ 2.36)	< 0.001	1.41 (1.29 ~ 1.53)	< 0.001
Model 1	2.21 (1.99 ~ 2.45)	< 0.001	1.42 (1.31 ~ 1.55)	< 0.001
Model 2	2.22 (2 ~ 2.46)	< 0.001	1.43 (1.32 ~ 1.56)	< 0.001
Model 3	1.43 (1.28 ~ 1.6)	< 0.001	1.16 (1.06 ~ 1.27)	< 0.001

Model 0: non-adjusted.

Model 1: age、sex、race.

Model 2: age、sex、race、income、marital status、residence.

Mode 3: age、sex、race、income、marital status、residence、site、grade、pT status.

### Subgroup analysis

**Fig. 4:**
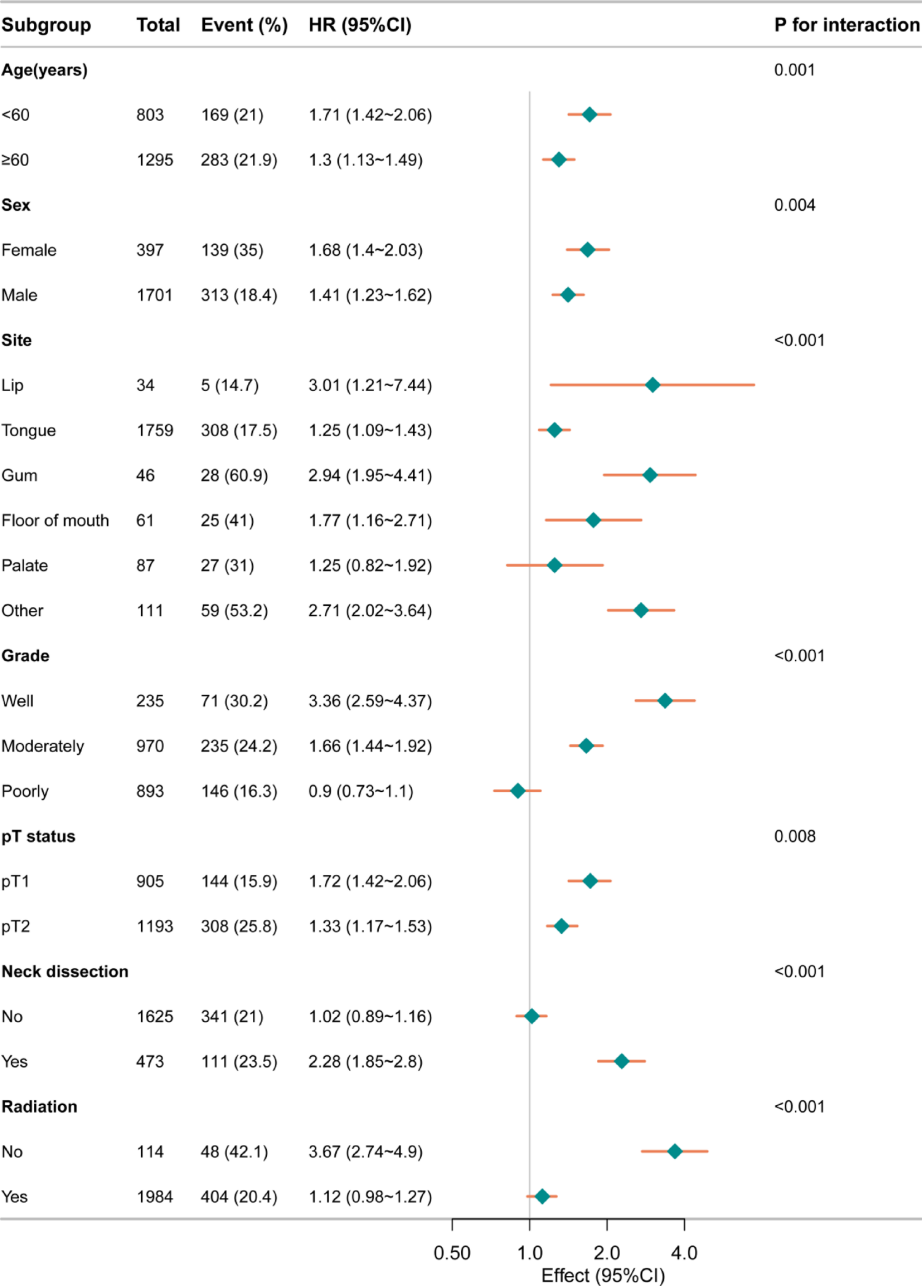
Forest plot illustrating the association between Adjuvant CT and DSS across various subgroups.

**Fig. 5:**
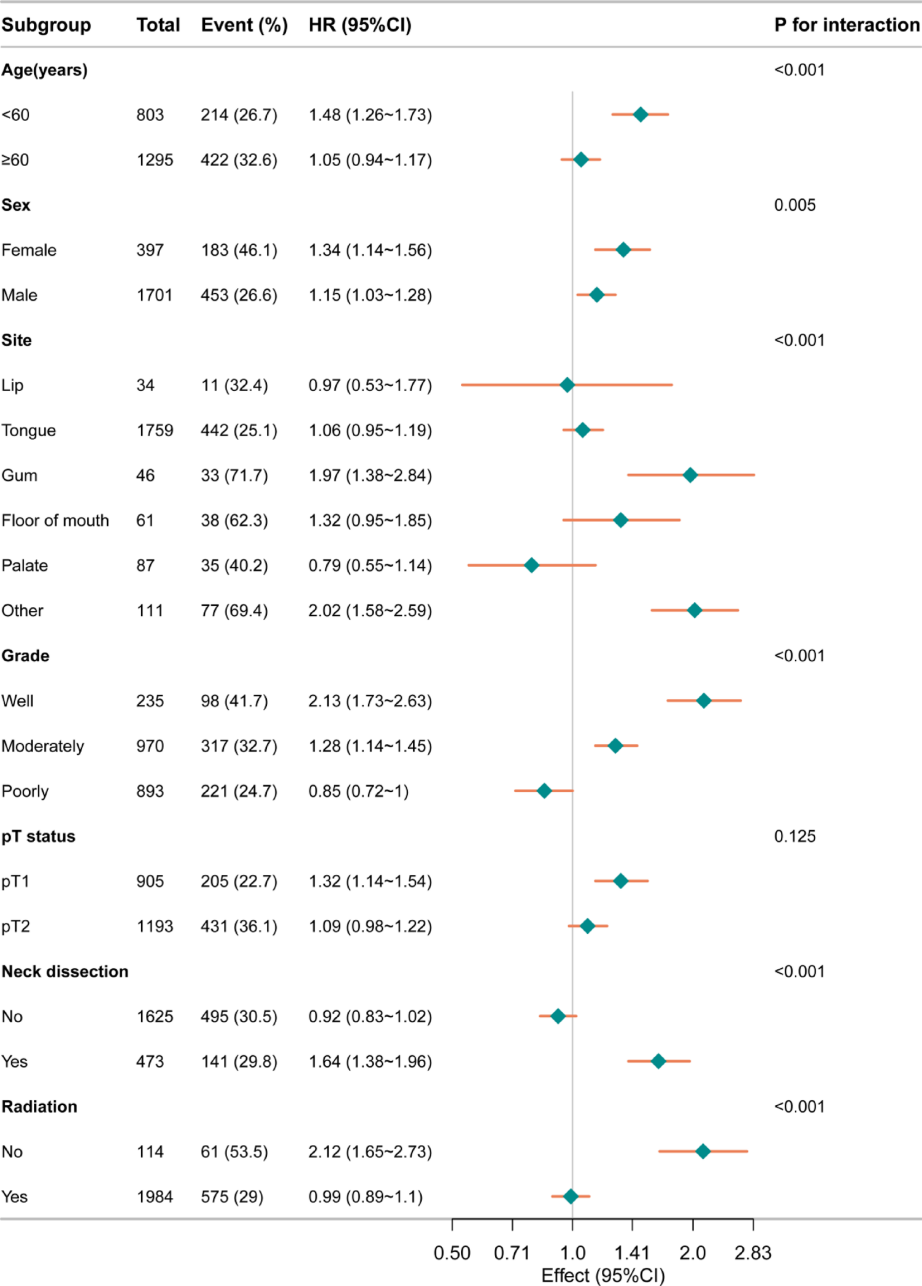
Forest plot illustrating the association between Adjuvant CT and OS across various subgroups.

Stratification and interaction analyses were conducted to evaluate the robustness of Adjuvant CT across various population characteristics. Forest plot analysis revealed significant variations in the HR for survival outcomes across different subgroups, indicating that the effect of Adjuvant CT may be modified by specific patient characteristics. In subgroup analyses of DSS, participants younger than 60 years exhibited a higher risk of event occurrence (HR:1.71, 95% Cl:1.42–2.06) compared to those aged 60 and older (HR:1.3, 95% Cl:1.13–1.49), with a significant interaction between age subgroup (*P* < 0.001). Similarly, female participants had a higher risk (HR:1.68,95%Cl:1.4–2.03) than male participants (HR:1.43,95%Cl:1.23–1.62), with a significant interaction between gender subgroup (*P* = 0.004). Tumor site significantly affected prognosis, especially in the gum (HR:2.94,95% Cl:1.95–4.41) and the lip (HR:3.01, 95% Cl:1.21–7.44). Regarding pathological grades, well differentiated tumors exhibited the highest risk (HR:3.36,95% Cl: 2.59–4.37), whereas poorly differentiated tumors demonstrated the lowest risk (HR: 0.9,95% Cl: 0.73–1.1). There were significant interactions observed among the grade subgroup (*P* < 0.001). The radiotherapy group exhibited a lower risk (HR:1.12,95%Cl:0.98–1.27) compared to the non-radiotherapy subgroup, with a significant interaction (*P* < 0.001) Similarly, in the pT status and the neck dissection subgroup, adjuvant CT was identified as a risk factor (Fig. 4). In subgroup analyses of OS, similar trends were observed. Participants younger than 60 years had a higher impact (HR:1.48,95%Cl:1.26–1.73) compared to those aged 60 and older 60 years (HR:1.05,95%Cl:0.94–1.17), with a significant interaction between age subgroup (*P* < 0.001). Females exhibited a higher HR (1.34,95%Cl:1.14–1.56) than males (HR:1.15,95%Cl:1.03–1.28)), with a significant interaction between sex subgroup (*P* = 0.005). Tumor site significantly impacted risk, such as the gum (HR: 1.97,95%Cl: 1.38 ~ 2.84) and the floor of the mouth (HR: 1.32, 95%Cl: 0.95–1.85). Among pathological grades, well differentiated tumors were associated with the highest risk (HR: 2.13, 95%Cl: 1.73–2.63), whereas poorly differentiated tumors had the lowest risk (HR:0.85, 95%Cl: (0.72-1), with significant interactions between these subgroups (*P* = 0.004). Patients receiving radiotherapy had a lower HR (0.99, 95%Cl:0.89–1.1) compared to those without, indicating a potential beneficial effect of radiotherapy on reducing risk (*P* < 0.001) (Fig. 5). Considering the interaction between adjuvant radiotherapy and adjuvant CT, as well as the fact that radiotherapy itself serves as a critical adjuvant treatment modality, we further stratified the study population into non-radiotherapy and radiotherapy groups to more comprehensively assess the efficacy of adjuvant CT. Overall, for patients with early-stage OSCC after surgery, compared with adjuvant radiotherapy, although adding chemotherapy on the basis of radiotherapy may improve the OS of patients to a certain extent (*P* = 0.029) (Fig. 2K), the results of non-radiotherapy groups suggest that adjuvant CT may become an unfavorable factor affecting prognosis in most cases (*P* < 0.0001) (Figs. 1K and 2K, and supp.Tables 1–4).

## Discussion

Early-stage OSCC generally has a favorable prognosis, the primary objective of treatment is to achieve complete tumor resection while preserving maximal oral function and aesthetics^22^. Surgical intervention remains the cornerstone of early-stage OSCC management, with radical excision being feasible in most cases^23^. However, despite optimal surgical treatment, a significant proportion of patients experience recurrence or mortality. The role of Adjuvant CT in improving prognosis remains a subject of ongoing debate.

This study evaluated the efficacy of postoperative adjuvant CT in early-stage OSCC patients. KM survival analysis revealed that postoperative adjuvant CT did not significantly enhance patient survival outcomes, including DSS and OS. In the Cox proportional hazards analysis, we meticulously accounted for potential confounding factors that may influence the postoperative prognosis of early-stage OSCC, including age, gender, and marital status, etc. These adjustments further ensured the robustness of our findings. After PSM, Adjuvant CT remained a still a risk factor for early-stage OSCC after postoperative, which confirmed the stability and reliability of our results. The results of the subgroup analysis were largely consistent. However, we observed a potential benefit of adjuvant CT in improving DSS and OS among the poorly differentiated subgroup. These findings have significant implications for refining treatment strategies for early-stage OSCC.

The primary mechanism may involve chemotherapy-induced alterations in the immune microenvironment of OSCC. In early-stage OSCC, the tumor microenvironment often exhibits a relatively “immune-active” phenotype, characterized by robust immune cell infiltration and heightened immune responses^24^. This condition supports the body’s innate immune surveillance mechanisms, potentially inhibiting tumor recurrence and metastasis^25^. However, while chemotherapy effectively targets tumor cells, it can also have detrimental effects on immune cells within the tumor microenvironment^26^. For instance, chemotherapy can induce apoptosis in immune cells, leading to functional impairment, or promote the expansion of immunosuppressive cells^27^. Such disruptions to the immune microenvironment compromise the body’s anti-tumor immune response, thereby undermining some of the potential benefits of chemotherapy^28^. Moreover, chemotherapy agents may alter the immunogenicity of tumor cells, enabling them to evade immune recognition and attack. Therefore, Adjuvant CT not only fails to enhance the immune defense mechanism in patients with early-stage OSCC, but it may also disrupt the normal functioning of the immune microenvironment. However, for patients with poorly differentiated early-stage OSCC, Adjuvant CT may improve clinical outcomes by inhibiting the proliferation and migration of cancer cells, which are more aggressive and have a higher propensity to metastasize^29,30^.

While the study yielded significant findings, several limitations should be acknowledged. First, the study did not encompass other critical pathological features, such as surgical margins, perineural invasion, vascular infiltration, and comorbidities. For example, the status of the surgical margin directly affects the risk of local recurrence and long-term survival in patients with early-stage OSCC^31^while the presence of perineural invasion and vascular invasion is associated with a higher risk of recurrence and metastasis^32,33^. In addition, comorbidities may affect the patient’s treatment tolerance and postoperative recovery^34^. Due to the absence of this information, we may not be able to accurately identify high-risk patients, thereby underestimating their risk of recurrence, which in turn affects the accurate assessment of overall prognosis. Future research should provide a more comprehensive analysis of these factors to enhance the understanding of OSCC progression and treatment outcomes. Second, this study utilized the SEER database for retrospective analysis, which may contain potential inaccuracies in some data points. We excluded patients with incomplete information and conducted subgroup analyses using OS and DSS as outcome measures to validate the robustness of our results and mitigate the potential biases associated with retrospective data. Due to insufficient data regarding postoperative CT regimens, the comprehensive assessment of the efficacy in patients with OSCC remains constrained. Future studies should leverage detailed chemotherapy databases or multi-center collaborations. Meanwhile, considering that immune checkpoint inhibitors have shown significant efficacy in the treatment of various solid tumors, the combined application of immunotherapy and conventional treatments (surgery, chemotherapy) should also be explored to improve the survival rate and quality of life of patients with early-stage OSCC. Finally, owing to the absence of tumor invasion depth (DOI) data in the SEER database, this study was unable to precisely stage the patients included between 2004 and 2017 according to the AJCC 8th edition criteria. Consequently, some pT3 tumors may be erroneously categorized as early-stage OSCC, potentially compromising the accuracy of the research findings. Moving forward, we plan to broaden our data sources, establish multi-center collaborations, and perform sensitivity analyses to enhance the precision of our research outcomes.

## Conclusion

Our study suggests that postoperative Adjuvant CT does not significantly enhance overall prognosis in early-stage OSCC. However, it may potentially benefit patients with poorly differentiated tumors. Therefore, a comprehensive evaluation of the necessity for Adjuvant CT, including an assessment of its potential benefits and risks, is essential for these patients.

## Methods

### Data sources

**Fig. 6:**
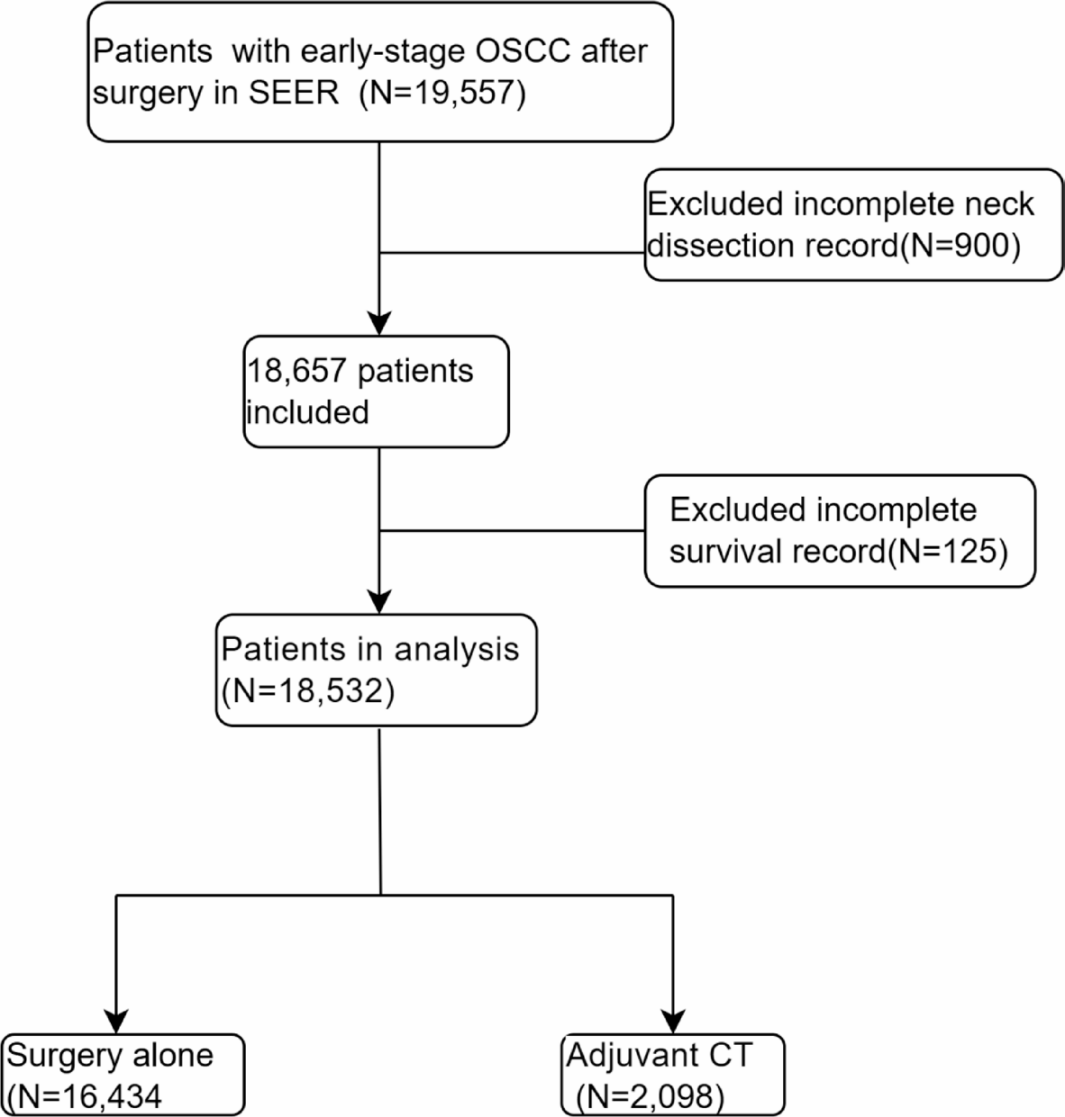
Flow chart of the study.

This large-scale retrospective cohort study utilized population-based data from the SEER database (SEER*Stat version 8.4.4), encompassing 17 registries, to examine patients diagnosed with primary early-stage OSCC between 2004 and 2021.The patients were categorized according to the staging criteria of the 7th edition of the AJCC classification (2004–2017) and the 8th edition (2018–2021). Baseline data for patients diagnosed with OSCC were extracted based on the International Classification of Diseases for Oncology, Third Edition (ICD-O-3) morphological codes: 8050–8076, 8078, 8083, 8084, and 8094. The exclusion criteria were as follows: (1) incomplete records of survival data (including DSS and OS); and (2) incomplete records of neck dissection (Fig. 6). Given that the data used in this study were publicly available, additional review by an ethics committee was not required.

#### Selection and definition of variables

All patients diagnosed with early-stage OSCC underwent surgical intervention and were subsequently stratified into two cohorts based on the administration of Adjuvant CT: surgery alone and Adjuvant CT. The SEER cause-specific death classification pertains to fatalities directly attributable to OSCC, denoted as disease-specific survival (DSS). In contrast, vital status refers to overall survival (OS). The prognostic indicators in the study included DSS and OS. Other important variables included age at diagnosis (< 60, and ≥ 60 years old), sex (male and female), the primary site of OSCC, grade (well(1), moderately (2), poorly (3) differentiated, and unknown), race (white, black, other (including American Indian/Alaska Native and Asian/Pacific Islander), and unknown), marital status (married, single, other, and unknown), residence(urban, and rural), income (<$50,000, $50,000–$75,000, and >$75,000), pT status(pT1,and pT2),neck dissection status (neck observation, and neck dissection), and radiotherapy.

We employed PSM to minimize bias between the two groups. The final cohort consisted of 3,186 patients, with 1,593 patients in each of the surgery alone and Adjuvant CT groups: The following covariates were matched: age, sex, site, grade, race, marital status, residence, income, pT status, neck dissection status, and radiation. Univariate analyses were conducted for both the entire cohort and the matched cohort. Multivariate analyses as well as subgroup analyses were performed on the unmatched cohort. We opted for the unmatched cohort over the matched cohort, as the process of matching might eliminate the association between the matched covariates and the outcome.

#### Statistical analyses

To compare the demographic and clinical characteristics of patients, various statistical methods were employed. Continuous variables that followed a normal distribution were summarized using mean ± standard deviation, while categorical variables were presented as frequency and percentage. For continuous variables with a normal distribution, inter-group differences were assessed using the independent samples t-test; for those not normally distributed, the Mann-Whitney U test was applied. Categorical variables were compared using the Chi-square test or Fisher’s exact test, as appropriate. The median follow-up period denotes the median duration from a patient’s enrollment in the study to their last follow-up. Survival analysis was performed using the Kaplan-Meier method, and independent prognostic factors were identified through multivariate Cox regression analysis. The robustness of the outcomes was further validated using Cox regression analysis. In this study, the missing values of covariates were addressed using a single imputation method. All statistical analyses were conducted using Free Statistics Software version 2.0 and R version 4.2.2. Statistical significance was defined as *P* < 0.05.

## Electronic supplementary material

Below is the link to the electronic supplementary material.

Supplementary Material 1

Supplementary Material 2

Supplementary Material 3

Supplementary Material 4

## Data Availability

Data will be made available on request (Should any party require access to the data from this study; they may contact Dr. Yungang He).
