# Supplementary material for: The effect of postoperative adjuvant chemotherapy on survival outcomes in patients with early stage oral squamous cell carcinoma
Source: Sci Rep. 2025 Jul 25;15:27157. doi: 10.1038/s41598-025-11565-y (PMC12297261; doi:10.1038/s41598-025-11565-y)
Supplement: Supplementary file 1 — Supplementary Material 1 [file 41598_2025_11565_MOESM1_ESM.docx]

| **Variables** | **Diseases-specific survival** | | **Overall survival** | |
| --- | --- | --- | --- | --- |
|  | **HR (95%CI)** | ***P-*value** | **HR (95%CI)** | ***P-*value** |
| **Age** |  |  |  |  |
| <60years | Ref. |  | Ref. |  |
| ≥60years | 2.03 (1.81,2.27) | < 0.001 | 3.37 (3.12,3.64) | < 0.001 |
| **Sex** |  |  |  |  |
| Female | Ref. |  | Ref. |  |
| Male | 0.82 (0.75,0.91) | < 0.001 | 0.97 (0.91,1.03) | 0.303 |
| **Tumor site** |  |  |  |  |
| Lip | Ref. |  | Ref. |  |
| Tongue | 3.62 (2.98,4.39) | < 0.001 | 0.98 (0.9,1.06) | 0.536 |
| Gum | 4.84 (3.79,6.19) | < 0.001 | 1.46 (1.29,1.65) | < 0.001 |
| Floor of mouth | 4.63 (3.67,5.84) | < 0.001 | 1.58 (1.42,1.76) | < 0.001 |
| Palate | 5.25 (3.86,7.13) | < 0.001 | 1.92 (1.64,2.25) | < 0.001 |
| Other | 5.3 (4.19,6.71) | < 0.001 | 1.49 (1.32,1.68) | < 0.001 |
| **Grade** |  |  |  |  |
| Well | Ref. |  | Ref. |  |
| Moderately | 2.07 (1.83,2.34) | < 0.001 | 1.34 (1.26,1.44) | < 0.001 |
| Poorly | 3.76 (3.22,4.38) | < 0.001 | 1.95 (1.77,2.15) | < 0.001 |
| **Race** |  |  |  |  |
| White | Ref. |  | Ref. |  |
| Black | 1.68 (1.32,2.14) | < 0.001 | 1.32 (1.12,1.56) | < 0.001 |
| Other | 0.9995 (0.8304,1.2031) | 0.996 | 0.72 (0.63,0.82) | < 0.001 |
| **Marital status** |  |  |  |  |
| Married | Ref. |  | Ref. |  |
| Single | 1.37 (1.19,1.57) | < 0.001 | 1.17 (1.07,1.28) | < 0.001 |
| Other | 2.04 (1.82,2.28) | < 0.001 | 2.19 (2.05,2.35) | < 0.001 |
| **Residence** |  |  |  |  |
| Urban | Ref. |  | Ref. |  |
| Rural | 0.98 (0.85,1.12) | 0.735 | 1.19 (1.1,1.29) | < 0.001 |
| **Income** |  |  |  |  |
| <50,000 | Ref. |  | Ref. |  |
| 50,000–75,000 | 0.77 (0.65,0.93) | 0.006 | 0.8 (0.71,0.89) | < 0.001 |
| >75,000 | 0.72 (0.6,0.86) | < 0.001 | 0.69 (0.62,0.77) | < 0.001 |
| **pT status** |  |  |  |  |
| pT1 | Ref. |  | Ref. |  |
| pT2 | 2.44 (2.2,2.7) | < 0.001 | 1.79 (1.68,1.92) | < 0.001 |
| **Neck dissection** |  |  |  |  |
| No | Ref. |  | Ref. |  |
| Yes | 0.91 (0.81,1.01) | 0.069 | 0.78 (0.73,0.83) | < 0.001 |
| **Adjuvant CT** |  |  |  |  |
| No | Ref. |  | Ref. |  |
| Yes | 5 (3.75,6.67) | < 0.001 | 2.54 (1.97,3.27) | < 0.001 |
