# Supplementary material for: The effect of postoperative adjuvant chemotherapy on survival outcomes in patients with early stage oral squamous cell carcinoma
Source: Sci Rep. 2025 Jul 25;15:27157. doi: 10.1038/s41598-025-11565-y (PMC12297261; doi:10.1038/s41598-025-11565-y)
Supplement: Supplementary file 2 — Supplementary Material 2 [file 41598_2025_11565_MOESM2_ESM.docx]

|  | **Disease- specific survival** | | **Overall survival** | |
| --- | --- | --- | --- | --- |
|  | **HR((95%CI)** | ***P-*value** | **HR((95%CI)** | ***P-*value** |
| **Model 0** | 5 (3.75~6.67) | <0.001 | 2.54 (1.97~3.27) | <0.001 |
| **Model 1** | 5.03 (3.77~6.71) | <0.001 | 2.47 (1.92~3.19) | <0.001 |
| **Model 2** | 4.9 (3.657~6.54) | <0.001 | 2.42 (1.88~3.12) | <0.001 |
| **Model 3** | 3.67(2.74~4.9) | <0.001 | 2.12 (1.65~2.73) | <0.001 |

**Model 0:** non-adjusted

**Model 1:** age、sex、race

**Model 2:** age、sex、race、income、marital status、residence

**Mode 3:**age、sex、race、income、marital status、residence、site、grade、pT status
