# Supplementary material for: The effect of postoperative adjuvant chemotherapy on survival outcomes in patients with early stage oral squamous cell carcinoma
Source: Sci Rep. 2025 Jul 25;15:27157. doi: 10.1038/s41598-025-11565-y (PMC12297261; doi:10.1038/s41598-025-11565-y)
Supplement: Supplementary file 3 — Supplementary Material 3 [file 41598_2025_11565_MOESM3_ESM.docx]

| **Variables** | **Diseases-specific survival** | | **Overall survival** | |
| --- | --- | --- | --- | --- |
|  | **HR (95%CI)** | ***P-*value** | **HR (95%CI)** | ***P-*value** |
| **Age** |  |  |  |  |
| <60years | Ref. |  | Ref. |  |
| ≥60years | 1.59 (1.41,1.8) | < 0.001 | 2.07 (1.87,2.29) | < 0.001 |
| **Sex** |  |  |  |  |
| Female | Ref. |  | Ref. |  |
| Male | 0.66 (0.58,0.74) | < 0.001 | 0.74 (0.67,0.81) | < 0.001 |
| **Tumor site** |  |  |  |  |
| Lip | Ref. |  | Ref. |  |
| Tongue | 2.54 (1.73,3.74) | < 0.001 | 1.11 (0.9,1.37) | 0.314 |
| Gum | 5.03 (3.27,7.74) | < 0.001 | 2.15 (1.66,2.79) | < 0.001 |
| Floor of mouth | 3.24 (2.12,4.95) | < 0.001 | 1.66 (1.3,2.11) | < 0.001 |
| Palate | 4.41 (2.88,6.74) | < 0.001 | 2.01 (1.56,2.58) | < 0.001 |
| Other | 4.61 (3.07,6.94) | < 0.001 | 2.09 (1.66,2.64) | < 0.001 |
| **Grade** |  |  |  |  |
| Well | Ref. |  | Ref. |  |
| Moderately | 0.94 (0.81,1.11) | 0.481 | 0.89 (0.79,1.01) | 0.077 |
| Poorly | 0.83 (0.69,0.99) | 0.035 | 0.81 (0.71,0.93) | 0.003 |
| **Race** |  |  |  |  |
| White | Ref. |  | Ref. |  |
| Black | 1.46 (1.17,1.82) | < 0.001 | 1.38 (1.15,1.65) | < 0.001 |
| Other | 1.13 (0.91,1.41) | 0.258 | 0.97 (0.81,1.16) | 0.725 |
| **Marital status** |  |  |  |  |
| Married | Ref. |  | Ref. |  |
| Single | 1.15 (0.98,1.35) | 0.097 | 1.12 (0.98,1.28) | 0.087 |
| Other | 1.55 (1.35,1.77) | < 0.001 | 1.76 (1.59,1.96) | < 0.001 |
| **Residence** |  |  |  |  |
| Urban | Ref. |  | Ref. |  |
| Rural | 1.14 (0.97,1.34) | 0.122 | 1.14 (1,1.3) | 0.045 |
| **Income** |  |  |  |  |
| <50,000 | Ref. |  | Ref. |  |
| 50,000–75,000 | 0.97 (0.78,1.2) | 0.765 | 0.94 (0.79,1.12) | 0.492 |
| >75,000 | 0.7 (0.56,0.86) | < 0.001 | 0.71 (0.6,0.85) | < 0.001 |
| **pT status** |  |  |  |  |
| pT1 | Ref. |  | Ref. |  |
| pT2 | 1.49 (1.32,1.69) | < 0.001 | 1.34 (1.22,1.47) | < 0.001 |
| **Neck dissection** |  |  |  |  |
| No | Ref. |  | Ref. |  |
| Yes | 0.71 (0.63,0.8) | < 0.001 | 0.63 (0.57,0.7) | < 0.001 |
| **Adjuvant CT** |  |  |  |  |
| No | Ref. |  | Ref. |  |
| Yes | 1.03 (0.91,1.16) | 0.655 | 0.9 (0.81,0.99) | 0.029 |
