# Supplementary material for: The effect of postoperative adjuvant chemotherapy on survival outcomes in patients with early stage oral squamous cell carcinoma
Source: Sci Rep. 2025 Jul 25;15:27157. doi: 10.1038/s41598-025-11565-y (PMC12297261; doi:10.1038/s41598-025-11565-y)
Supplement: Supplementary file 4 — Supplementary Material 4 [file 41598_2025_11565_MOESM4_ESM.docx]

|  | **Disease- specific survival** | | **Overall survival** | |
| --- | --- | --- | --- | --- |
|  | **HR((95%CI)** | ***P-*value** | **HR((95%CI)** | ***P-*value** |
| **Model 0** | 1.03 (0.91~1.16) | 0.655 | 0.9 (0.81~0.99) | 0.029 |
| **Model 1** | 1.1 (0.97~1.25) | 0.127 | 0.94 (0.85~1.04) | 0.199 |
| **Model 2** | 1.11 (0.98~1.26) | 0.09 | 0.95 (0.86~1.05) | 0.29 |
| **Model 3** | 1.12 (0.98~1.27) | 0.094 | 0.99 (0.89~1.1) | 0.86 |

**Model 0:** non-adjusted

**Model 1:** age、sex、race

**Model 2:** age、sex、race、income、marital status、residence

**Mode 3:**age、sex、race、income、marital status、residence、site、grade、pT status
